# Further validation of the Gothenburg Trismus Questionnaire (GTQ)

**DOI:** 10.1371/journal.pone.0243805

**Published:** 2020-12-17

**Authors:** Mia Johansson, Therese Karlsson, Caterina Finizia

**Affiliations:** 1 Department of Oncology, Institute of Clinical Sciences, Sahlgrenska Academy, Gothenburg University, Gothenburg, Sweden; 2 Department of Otorhinolaryngology, Region Västra Götaland, Sahlgrenska University Hospital, Gothenburg, Sweden; 3 Department of Otorhinolaryngology, Head and Neck Surgery, Institute of Clinical Sciences, Sahlgrenska Academy, Gothenburg University, Gothenburg, Sweden; Iranian Institute for Health Sciences Research, ISLAMIC REPUBLIC OF IRAN

## Abstract

This study aimed to update and, if necessary, revise the Gothenburg Trismus Questionnaire (GTQ), the only existing trismus-specific questionnaire, and retest its psychometric properties. Semi-structured interviews were performed with 10 trismus patients of which 5 had head and neck cancer (HNC) and 5 suffered from benign temporomandibular disorders. Trismus was defined as a maximal incisal opening of ≤ 35mm. An expert panel discussed and revised the GTQ based on interview information, expertise knowledge and the original questionnaire. The revised questionnaire was then tested in a study sample consisting of benign jaw-related conditions (n = 26), patients treated for HNC (n = 90) and an age- and gender-matched control group with no trismus (n = 116). The revised version of the GTQ (GTQ 2) was well accepted by patients. The original three domains continued to show high internal consistency (Cronbach’s alpha 0.74–0.94) and construct validity. Two dually posed single items were split into four questions and the wording was altered in another three items. Moreover, a new domain (Facial pain) was identified, which had excellent internal consistency (α = 0.96) and good construct validity. The revision of the original Gothenburg Trismus Questionnaire (GTQ 1) with inclusion of patient-input, resulted in splitting of ambiguous items, identifying a fourth domain named *Facial pain* and the recall time shortened for some items. Additionally, the remaining domains and items were re-confirmed as strong in the psychometric analysis. Henceforth, the new version, GTQ 2 should be used.

## Introduction

Trismus is defined as a limitation in the ability to open the mouth or jaw caused by reduced mandible motility. In objective measures, trismus is seen when the maximal incisal opening (MIO) is ≤ 35 mm [1]. The condition may be caused by benign jaw related disorders (temporomandibular disorders, TMD) or by local and metastatic head and neck tumors as well as a result of the oncological treatment, foremost radiotherapy [2]. It is a painful condition that impacts negatively on the patient’s quality of life as trismus affects food intake, oral hygiene and social contacts [3, 4].

In health care today, patient-reported outcome instruments are an established complement to objective outcome measures. For this purpose, the Gothenburg Trismus Questionnaire (GTQ 1) was developed in 2012 serving as the first comprehensive trismus-specific questionnaire covering different aspects of trismus, to be used as a complement to the objective measure MIO [1, 5]. The GTQ can be used both in clinical care, e.g. to chart the patients bother of trismus or to evaluate rehabilitation interventions and in clinical research on trismus. To date, the GTQ 1 is also being translated into several languages including English and Portugese.

When the GTQ 1 was first developed and validated, two important psychometric aspects were included, namely testing of reliability and validity. The aforementioned concept investigates the reliability of an instrument’s measurements as well as its ability to reproduce the measures data-points. This is often done using Cronbach’s alpha and the Intraclass correlation coefficient, both in which GTQ 1 scored well [5]. Validity, on the other hand, ensures that the instrument measures what it is intended to measure. Firstly, content validity describes how the content for the items was generated, whilst construct validity refers to how effectively the items measure the latent variable. Finally, criterion validity compares the domains in the new instrument to similar domains in external instruments, which again assures that the novel instrument captures relevant aspects of the desired variable [6].

During the development of GTQ 1, patient input was not included until the confirmatory phase as opposed to in the very initial stages of the item generation. During its utilization, there has also been some ambiguity regarding dually posed items. Hence, this study aims to update and, if necessary, revise the GTQ and retest its psychometric properties.

## Material and methods

### Patient interviews

In order to increase patients’ involvement in the content and design of the GTQ instrument, semi- structured face-to-face interviews were performed with trismus patients. All interviews were conducted by the first author who was unknown to the patients and not involved in their care. Patients were asked, in open-ended questions, to describe their symptoms and problems associated with trismus. The interviews were tape-recorded and transcribed verbatim by the first author. The information retrieved from the interviews was analyzed by the use of content analysis [7]. The interviews were performed until the data was matured, i.e., no additional information came out.

### Expert panel

Following the interviews an expert panel consisting of dentists, a stomatognathic physiologist, otolaryngologists, an oncologist and a behaviourist was organized. The panel discussed, in-depth, the information derived from the interviews as well as the content and construction of the original GTQ. Based on the outcome of the discussion, the GTQ instrument was revised.

### Cognitive debriefing

After revision of the GTQ instrument, individual interviews were conducted to confirm the relevance and interpretability of the items. Patients were administered the revised GTQ instrument draft and were thereafter asked how items were perceived or whether patients found any items to be missing.

### Confirmatory psychometric validation phase

#### Participants

Patients with head and neck cancer (HNC) at risk of developing trismus were identified and considered eligible for the study at the weekly multidisciplinary board review at the department of Otorhinolaryngology at Sahlgrenska University Hospital, Gothenburg, Sweden. Patients with temporomandibular disorders (TMD) were included at regular visits to a stomatognathic physiologist at the Institute of Odontology, Sahlgrenska academy, Gothenburg. The inclusion period lasted between June 2018 and April 2019.

Patients with poor language comprehension and cognition were considered non-eligible. Instruments were distributed to patients at the clinics and mailed back. Patients who had not returned their instruments within 2 weeks were reminded once.

The desired sample size was 115 participants in accordance to guidelines by Fayers and Machin where five respondents per item are required [6].

The study also comprised an age- and gender-matched control group of patients from the department of Otorhinolaryngology at Sahlgrenska University Hospital. The controls denied trismus and had no clinical evidence of such and completed the GTQ instrument in clinic.

#### Construct validity

The Spearman correlation coefficient was used to evaluate convergent and discriminant validity. For assessment of convergent and discriminant validity the GTQ domains was compared to the domains of the European Organization for Research and Treatment of Cancer Quality of Life Questionnaire, Core Module (QLQ-C30) and Head and Neck Module (QLQ-H&N35) as well as the Hospital Anxiety and Depression Scale (HADS). Known-group validity was assessed by comparing GTQ domain scores between patients with and without trismus as well as with the control group.

#### Reliability

Reliability of the GTQ domains was assessed with Cronbach’s alpha coefficient where an alpha value > 0.7 was considered as supporting internal consistency reliability [6]. Test-retest reliability was assessed in 29 of the patients between inclusion and two weeks later, when they were administered the GTQ once more. Test-retest reliability was assessed using Intra-class Correlation Coefficients (ICC), with ICC values between 0.4–0.75 considered to represent fair to good reliability and values > 0.75 excellent reliability [6].

### Patient reported outcome measures

#### Original Gothenburg Trismus Questionnaire (GTQ 1)

The GTQ version 1 contains 21 items; with 13 items divided into the 3 domains: Jaw-Related Problems (6 items), Eating Limitation (4 items), and Muscular Tension (3 items). The remaining 8 items are retained as single items. The domains and single items score range from 0 to 100, in which 100 indicate the maximal amount of symptoms and 0 is equal to no symptoms. The recall period is one week for domains and vary between one week and one month for single items [5]

#### EORTC QLQ-C30 and QLQ-H&N3

The EORTC QLQ-C30 assesses the physical and psychosocial functioning and symptom experiences of cancer patients in general. To address additional symptoms associated specifically with HNC and its treatment, the complementary 35-item module can be used, the QLQ-H&N35. The core instrument [8] as well as the HNC-specific module [9] have demonstrated satisfactory to excellent reliability and validity. Recall periods for both instruments are one week.

#### Hospital Anxiety and Depression Scale

The Hospital Anxiety and Depression Scale (HADS) is a validated scale, used to detect mood disorders in patients with somatic comorbidity [10]. HADS contains 14 questions and the score is divided into a depression score and an anxiety score, each ranging from 0–21. The range is divided into scores of 0–7, 8–10 and >10 corresponding to no problems, possible and probable anxiety/depression respectively. The recall period is one week.

### Statistical analysis

Descriptive statistics were calculated according to standard procedures. For pairwise comparison between groups, Fisher´s Exact test for dichotomous variables was applied and the Mantel-Haenszel Chi Square Exact test was applied for ordered categorical variables and the Fishers Permutation Test for continuous variables. All tests were two-tailed and the significance level set to 5% throughout.

Cohens conventions was used to interpret effect sizes, where a correlation coefficient of 0.10 is thought to represent a weak or small association; a correlation coefficient of 0.30 is considered a moderate correlation; and a correlation coefficient of 0.50 or larger is thought to represent a strong or large correlation [11]. Factor analysis was performed with varimax rotation and factors with an eigenvalue >1 were retained [6].

All statistical analyses were performed using the statistical software SAS® System version 9.4 (SAS Institute Inc., Cary, NC, USA) (if not stated otherwise).

### Ethical considerations

The study was approved by the Regional Ethical Review Board at Gothenburg University and performed in accordance with the Declaration of Helsinki. All participants gave their written informed consent. Permission was obtained from the owners of the original questionnaire prior to validation and modification of the questionnaire.

## Result

### Patient interviews

Semi-structured interviews were performed with 10 patients with trismus, five previously treated for HNC and five with TMD. Interview participants were between 23 and 74 years old and the median time for duration of trismus was two years (range 3 months to 8 years). Most common symptoms described were difficulties to open the mouth, pain, noises from the jaw, stiffness in the yaw and pressing of the tongue. Several problems associated with eating were mentioned, such as difficulties putting food and cutlery in the mouth, difficulties eating solid foods and trouble biting off.

### Modifications of the GTQ

Based on the results of the patient interviews and the discussions during the expert panel, minor revisions of the GTQ instrument were made. Item number1 “During the last week, have you felt Fatigue/ stiffness in your jaw?” was divided in two separate questions, i.e. “During the last week, have you felt fatigue in your jaw?” and “During the last week, have you felt stiffness in your jaw?”. Item number 2 “During the last week, have you felt aches or pain in your face and jaw?” was correspondingly divided into two items. Item 5, “During the last week have you felt pain or soreness in your jaw muscles” was changed into “During the last week have you felt pain moving your jaw (opening mouth/chewing)”. Item number 14 was rephrased, from “How strong facial pain do you have right now?” to “How strong was the worst facial pain you have experienced during the last 24 hours?” Item number 19 was similarly rephrased from “How limited are you in your ability to open your mouth right now?” to “How limited has your ability to open your mouth been during the last 24 hours?” For the single items, the recall period was changed from one month to one week. Furthermore, a pain assessment picture was added, depicting the face divided into 12 areas, where patients can mark the location of the pain.

### Cognitive debriefing

After revision of the GTQ, eight patients filled out the instrument and were interviewed. The following interviews showed that the new GTQ 2 instrument was well accepted and the items easy to understand.

### Factor structure of the revised GTQ

Factor analysis with varimax rotation confirmed the three domains of the original GTQ version, but with the addition of a fourth domain. The additional domain consists of five items; “How strong was the worst facial pain you have experienced during the last 24 hours?”, “How strong was the worst facial pain you have experienced during the last week?”, “On average, how strong has your facial pain been during the last week?”, “How much has your facial pain interfered with your social, leisure and family activities?” and “How much has your facial pain affected your ability to work (including both gainful employment and household duties)? This new domain was named *Facial pain*. The remaining three items were retained as single items as well as the six questions related to any kind of training to improve mouth opening.

### Confirmatory psychometric validation phase

#### Participants

Socio-demographic and clinical characteristics of the study participants and controls are found in Table 1. As expected, patients with HNC were significantly older than the TMD patients and the number of females were significantly higher in the TMD group. There was no difference in age and sex between the study group and controls. For the HNC group, most common tumor location was tonsil and for the TMD patients the most common diagnosis was disc related problems.

**Table 1:** Socio-demographic and clinical characteristics of the study participants and controls.

	Study group			Study group	Controls	
	HNC	TMD		HNC + TMD		
	Mean (SD)		p-value	Mean (SD)		p-value
	Median (Range)			Median (Range)		
**Age** (years)	67 (7.5)	45 (16.7)	< 0.0001	62 (13.9)	62 (13.1)	0.56
	67 (49–84)	45 (16–71)		65 (16–84)	65 (16–88)	
**MIO** (mm)	40.1 (10.3)	36.3 (8.1)	0.047	39.2 (9.9)	52.5 (6.8)	
		36 (20–50)		40 (16–58)	52 (37–73)	< 0.0001
	41 (16–58)					
**Gender**	n (%)			n (%)		
Male	68 (76)	4 (15)		72 (62)	72 (62)	
Female	22 (24)	22 (85)	< 0.001	44 (38)	44 (38)	1.00
**Marital status**						
Single	67 (74)	21 (81)		88 (76)	89 (77)	
Married/Cohabitant	23 (26)	5 (19)	0.70	28 (24)	26 (23)	0.91
**Education level**						
Elementary school	21 (24)	3 (12)		24 (21)	15 (13)	
High school	29 (33)	9 (38)		38 (34)	40 (35)	
University	38 (43)	12 (50)	0.31	50 (45)	59 (52)	0.12
**Occupational status**						
Working	24 (27)	12 (46)		36 (31)	50 (43)	
Retired	64 (71)	7 (27)		71 (61)	63 (54)	
Studying	0 (0)	5 (19)		5 (4)	3 (3)	
Other	2 (2)	2 (8)	< 0.001	4 (4)	0 (0)	0.064
**Smoking**						
Never smoked	39 (43)	16 (61)		55 (47)	63 (54)	
Stopped smoking	47 (52)	8 (31)		55 (47)	46 (40)	
Current smoker	4 (4)	2 (8)	0.26	6 (6)	7 (6)	0.44
**Cancer location**						
Tonsil	45 (50)					
Base of tongue	20 (23)					
Tumour colli	8 (9)					
Tongue	3 (3)					
Oropharynx	3 (3)					
Other	11 (12)					
**Reason for TMD**						
Disc problems		14 (54)				
Arthritis		6 (23)				
Muscular		6 (23)				

HNC = Head and neck cancer, TMD = temporomandibular disorders, MIO = Maximum incisal opening.

#### Reliability

The four domains showed good internal consistency (Cronbach’s alpha >0.70) and test-retest reliability was excellent (Table 2).

**Table 2:** Reliability estimates.

GTQ	Internal consistency^a^	Test-retest^b^
		ICC
Jaw related problems	0,94	0.98
Eating limitation	0,87	0.97
Muscular tension	0,74	0.95
Facial pain	0,96	0.98

^a^ Cronbach’s alpha.

^b^ Test-retest sample size n = 29.

ICC = Intra-class correlation coefficient.

#### Convergent and discriminant validity

Overall, the results of the correlation analyses confirmed the pre-specified hypotheses based on the results from the validation of the GTQ 1. Both moderate and strong correlations were found between the GTQ domains and some of the EORTC domains, see Table 3. There were strong correlations between the domain *Jaw related problems* and the *Pain* domains of both EORTC instruments as well as the domains *Opening mouth* and *Social eating* of the EORTC QLQ-HN35. The scores of the *Eating limitation* domain correlated strongly with three of the domains in the EORTC QLQ-HN35; *Pain*, *Social eating* and *Opening mouth*. For *Muscular tension* there were no strong correlations to any of the EORTC domains and for many of the domains, such as *senses*, *speech* and *sticky saliva* the correlations were weak. For the *Facial pain* domain of the GTQ there was a strong association with the domains *emotional function* and *Pain* of the EORTC QLQ-C30, as well as with the domains *Pain* and *Opening mouth* in the EORTC QLQ-HN35.

**Table 3:** GTQ scale correlations with EORTC QLQ C30 and H&N35 domains in HNC patients and TMD patients.

GTQ Domains	Jaw related problems	Eating limitation	Muscular tension	Facial pain
**EORTC QLQ-C30**	**r (p-value)**			
Physical Functioning	-0.19 (0.039)	-0.25 (0.007)	-0.13 (0.16)	-0.30 (<0.001)
Role Functioning	-0.36 (<0.001)	-0.34 (<0.001)	-0.14 (0.14)	-0.43 (<0.001)
Emotional Functioning	-0.47 (<0.001)	-0.35 (<0.001)	-0.45 (<0.001)	-0.53 (<0.001)
Cognitive Functioning	-0.39 (<0.001)	-0.35 (<0.001)	-0.28 (<0.001)	-0.43 (<0.001)
Social Functioning	-0.35 (<0.001)	-0.38 (<0.001)	-0.13 (0.18)	-0.47 (<0.001)
Global QoL	-0.38 (<0.001)	-0.35 (<0.001)	-0.23 (0.011)	-0.37 (<0.001)
Fatigue	0.37 (<0.001)	0.30 (0.001)	0.30 (0.001)	0.30 (<0.001)
Nausea and vomiting	0.29 (0.02)	0.23 (0.015)	0.24 (0.01)	0.46 (<0.001)
Pain	0.55 (<0.001)	0.39 (<0.001)	0.38 (<0.001)	0.54 (<0.001)
**EORTC QLQ-H&N35**				
Pain	0.74 (<0.001)	0.64 (<0.001)	0.39 (<0.001)	0.58 (<0.001)
Swallowing	0.23 (0.02)	0.34 (<0.001)	-0.09 (0.33)	0.15 (0.12)
Senses	0.07 (0.45)	0.07 (0.50)	0.03 (0.78)	0.07 (0.46)
Speech	0.21 (0.02)	0.28 (0.003)	0.05 (0.56)	0.24 (0.01)
Social eating	0.57 (<0.001)	0.69 (<0.001)	0.20 (0.04)	0.42 (<0.001)
Social contact	0.35 (<0.001)	0.37 (<0.001)	0.18 (0.047)	0.46 (<0.001)
Sexuality	0.21 (0.03)	0.21 (0.03)	0.10 (0.27)	0.16 (0.1)
Teeth	0.31 (<0.001)	0.26 (0.006)	0.12 (0.22)	0.24 (0.01)
Opening mouth	0.76 (<0.001)	0.72 (<0.001)	0.33 (<0.001)	0.53 (<0.001)
Dry mouth	0.04 (0.69)	0.11 (0.24)	0.05 (0.63)	-0.03 (0.74)
Sticky saliva	0.49 (0.60)	0.19 (0.04)	0.03 (0.76)	-0.01 (0.87)
Coughing	0.12 (0.22)	0.32 (<0.001)	0.14 (0.15)	0.03 (0.76)
Feeling ill	0.37 (<0.001)	0.31 (0.001)	0.28 (0.003)	0.44 (<0.001)

Spearman correlation coefficient (p-value); n = 116 (HNC + TMD).

The correlation analyses between the GTQ and the HADS are found in Table 4. The correlations were mainly moderate, with the strongest correlation (r = 0.48) between the GTQ domain *Facial pain* and the HADS domain *Anxiety*.

**Table 4:** GTQ scale correlations with HADS in HNC patients and TMD patients.

GTQ Domains	Jaw related problems	Eating limitation	Muscular tension	Facial pain
	**r (p-value)**			
**HADS Anxiety**	0.43 (<0.001)	0.32 (<0.001)	0.45 (<0.001)	0.48 (<0.001)
**HADS Depression**	0.39 (<0.001)	0.37 (<0.001)	0.26 (<0.001)	0.37 (<0.001)

Spearman correlation coefficient (p-value); n = 116 (HNC + TMD).

Known-group validity is shown in Table 5. For the HNC patients, the GTQ could discriminate between trismus and non-trismus patients, even if the difference was not statistically significant for the domain muscular tension. For the TMD patients, patients with trismus (MIO < 36 mm) scored higher (i.e. had more trismus related problems) in three of the domains compared to the patients with no trismus, but the difference was not statistically significant, except for the domain “jaw related problems”. Furthermore, HNC patients reported as expected significantly lower scores on all domains compared to TMD patients. There were also statistically significant differences when comparing the GTQ scores of the controls and the HNC patients and TMD patients respectively, even when including all patients and not only those with MIO < 36 mm.

**Table 5:** Mean GTQ scores for trismus and non-trismus patients.

	HNC	HNC		TMD	TMD		Controls	Controls *vs*.	Controls *vs*.
	MIO	MIO		MIO	MIO				
	>35 mm	< 36 mm		> 35 mm	< 36 mm			HNC*	TMD**
	Mean	Mean	p-value	Mean (SD)	Mean (SD)	p-value	Mean (SD)	p-value	p-value
	(SD)	(SD)							
Jaw related problems	14.8	34.5	< .0.0001	32.4 (11.7)	63.0 (28.5)	0.009	1.4 (2.91)	< .0.0001	< .0.0001
	(18.0)	(21.1)							
Eating limitation	13.0	37.3	< .0.0001	25.0 (21.3)	41.8 (30.9)	0.16	0.3	< .0.0001	< .0.001
	(21.9)	(25.8)					(1.6)		
Muscular tension	18.7	23.9	0.29	55.8 (22.1)	42.9 (27.6)	0.21	9.6	< .0.0001	< .0.0001
	(20.6)	(22.6)					(14.3)		
Facial pain	6.7	17.1	0.002	21.5 (16.0)	37.3 (34.3)	0.22	0.1	< .0.0001	< .0.0001
	(14.1)	(21.8)					(1.4)		

GTQ scoring: 0 indicating the most favourable state and 100 indicating the least favourable.

* All HNC patients, regardless of MIO-value.

** All TMD patients, regardless of MIO-value.

## Discussion

The original GTQ (GTQ 1) was developed due to the lack of a trismus-specific questionnaire and is in clinical use today, where it has been translated into several languages. However, due to the lack of original patient input and dually posed questions, a revision and update was needed, which was the aim of this study.

In general, the original three domains of GTQ 1 were re-confirmed as highly relevant. This was firstly seen by the new inclusion of patient input in the initial testing adding to content validity–an aspect that was lacking in the development of the original questionnaire. Secondly, both the domains *Jaw related problems* and *Eating limitation* showed strong correlations with hypothesized domains on the EORTC QLQ-C30 and EORTC QLQ-HN35, again emphasizing their construct validity. Additionally, all three original domains had even higher (and excellent) scores for internal consistency in this re-validation as opposed to the initial psychometric testing [5].

The factor analysis also confirmed the above mentioned three original domains but with the addition of a new fourth domain, named *Facial pain*, consisting of five items. This new domain appears to not only have excellent internal consistency (α = 0.96) but also relevant and strong construct validity when correlations were calculated against EORTC QLQ-C30 and EORTC QLQ-HN35, with correlations in excess of 0.50 for both pain and opening mouth items on the anchor EORTC questionnaires.

Interestingly, the *Facial pain*-domain also showed a strong correlation with the domain *Emotional functioning* on EORTC QLQ-C30 as well as with the *HADS Anxiety domain*, possibly indicating that pain is associated with mental health.

As seen in the original GTQ, the *Muscular tension* domain shows weak or moderate correlations with the EORTC. This may potentially be explained by the EORTC questionnaires being aimed at a populations of cancer patients, whereas muscular tension is a symptom mainly reported by the TMD-patients, and not by the HNC-patients, in the study (42.9 vs 23.9 respectively). A further explanation may be the constitution of the two study groups, namely that there were significantly younger patients in the TMD-group and more were female compared to the HNC group who also had suffered from trismus for a longer time-period. The weaker correlations of the *Muscular tension* domain may also be related to the fact that the domain captures muscular facial- and jaw problems in general, which is an important complement to the remaining domains. A study by Galves-Sanchez et al recently showed that pain experienced by patients with chronic pain conditions may increase both depression and anxiety [12]. Hence, the muscular tension experienced by TMD patients could well be considered a chronic pain variant as the only moderate correlations seen in this domain were with domains *HADS Anxiety* and EORTC domains ***Emotional*** *functioning*, *Pain and opening mouth*.

The recall period for all items and domains was changed to the last week, resulting in the same time frame of recall being used throughout the entire questionnaire. The prior use of a one month recall time was based on the theory that some items are less likely to fluctuate over time [13]. However, this was not necessarily the case and it caused confusion for patients who often neglected to notice a change in the stated recall period between different items.

The updated GTQ, GTQ 2 (S1 Appendix), now contains 29 items of which 20 items are divided into four domains; Jaw related problems (8 items), Eating limitations (4 items), Muscular tension (3 items) and Facial pain (5 items). It also includes 3 single items about mouth opening and 6 questions related to any kind of training to improve mouth opening. The new GTQ 2 also includes a picture of a face giving the patient the possibility to locate the pain. The domains are scored by calculating the mean of each domain and transforming it to a scale of range 0 to 100, where a maximum score indicates greater symptom burden due to trismus. It has now been tested in a patient sample that is representative for those with HNC or TMD, in terms of discriminating between those with trismus and those without. It can be accessed, free of charge, at http://entq.org, where permission can be sought for its use in clinical or research settings.

A strength of the study is the new inclusion of open, blinded patient-input, which only further confirmed the items already included in the original questionnaire. A limitation is the inability to assess responsiveness over time due to the cross-sectional design.

## Conclusion

The revision of the original Gothenburg Trismus Questionnaire (GTQ 1) resulted in splitting of ambiguous items, identifying a fourth domain named *Facial pain* and the recall time shortened. Additionally, the remaining domains and items were re-confirmed as strong in the psychometric analysis. Henceforth, the new version, GTQ 2 should be used.

## Supporting information

S1 Dataset (XLSX)

S1 Appendix (PDF)
