# Correction: Further validation of the Gothenburg Trismus Questionnaire (GTQ)

**DOI:** 10.1371/journal.pone.0245106

**Published:** 2020-12-31

**Authors:** Mia Johansson, Therese Karlsson, Caterina Finizia

## Notice of republication

An incorrect version of S1 Dataset was published in error. This article was republished on December 22, 2020, to correct for this error. Please download this article again to view the correct version.
