# Supplementary material for: Further validation of the Gothenburg Trismus Questionnaire (GTQ)
Source: PLoS One. 2020 Dec 17;15(12):e0243805. doi: 10.1371/journal.pone.0243805 (PMC7746186; doi:10.1371/journal.pone.0243805)
Supplement: S1 Appendix — (PDF) [file pone.0243805.s002.pdf]

Gothenburg Trismus Questionnaire (GTQ 2) *Items 1-23; if training 24-29*

This questionnaire contains questions related to mouth opening and jaw related problems. Please read each question carefully and answer by marking the alternative that best applies to you. Answer all questions and mark only one alternative for each question.

During the **last week,** have you:

		Not at all	Mild	Moderate	Severe	Very severe
1.	Felt fatigue in your jaw	☐1	☐2	☐3	☐4	☐5
2.	Felt stiffness in your jaw	☐1	☐2	☐3	☐4	☐5
3.	Felt pain in your face	☐1	☐2	☐3	☐4	☐5
4.	Felt pain in your jaw	☐1	☐2	☐3	☐4	☐5
5.	Felt pain moving your jaw (opening mouth/chewing)	☐1	☐2	☐3	☐4	☐5
6.	Had problems when opening your mouth wide or taking a big bite	☐1	☐2	☐3	☐4	☐5
7.	Felt pain in your jaw muscles	☐1	☐2	☐3	☐4	☐5
8.	Had problem yawning	☐1	☐2	☐3	☐4	☐5
9.	Had noises from your jaw	☐1	☐2	☐3	☐4	☐5

Due to your jaw problems, to what extent are you limited or incapable of:

		Not at all	Mild	Moderate	Severe	Very severe
10.	Eating solid food	☐1	☐2	☐3	☐4	☐5
11.	Putting food in mouth	☐1	☐2	☐3	☐4	☐5
12.	Eating soft food	☐1	☐2	☐3	☐4	☐5
13.	Bite off	☐1	☐2	☐3	☐4	☐5

Do you usually:

		Not at all	Seldom	Sometimes	Often	Very often
14.	Clench your teeth	☐1	☐2	☐3	☐4	☐5
15.	Press with your tongue	☐1	☐2	☐3	☐4	☐5

Questions 16-20 are about facial pain
By facial pain, we mean pain in the face and/or jaw related to your mouth opening ability

		No facial pain	Mild	Moderate	Severe	Very severe
16.	How strong was the worst facial pain you have experienced during ***the last 24 hours?***	☐1	☐2	☐3	☐4	☐5
17.	How strong was the worst facial pain you have experienced during the **last week**?	☐1	☐2	☐3	☐4	☐5
18.	On average, how strong has your facial pain been during the **last week**?	☐1	☐2	☐3	☐4	☐5

	During the **last week**	Not at all	A little	Moderately	Quite a bit	Very much
19.	How much has your facial pain interfered with your social, leisure and family activities?	☐1	☐2	☐3	☐4	☐5
20.	How much has your facial pain affected your ability to work (including both gainful employment and household duties)?	☐1	☐2	☐3	☐4	☐5

	Questions about mouth opening ability	Not at all	A little	Moderately	Quite a bit	Very much
21.	How limited has your ability to open your mouth been during ***the last 24 hours?***	☐1	☐2	☐3	☐4	☐5

	During the **last week**	No mouth opening limitation	A little	Moderately	Quite a bit	Very much
22.	How much has your mouth opening limitation interfered with your social, leisure and family activities?	☐1	☐2	☐3	☐4	☐5
23.	How much has your mouth opening limitation affected your ability to work (including both gainful employment and household duties)?	☐1	☐2	☐3	☐4	☐5

Mark the parts of the face where you feel any facial pain. You can select several options.
If you do not experience any facial pain, skip this image.

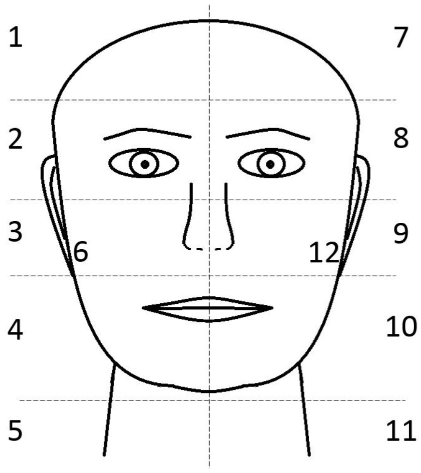

**Right side of the face**		**Left side of the face**	
1. Forehead right	☐	7. Forehead left	☐
2. Around the right eye	☐	8. Around the left eye	☐
3. Middle face right	☐	9. Middle face left	☐
4. Mouth/chin right	☐	10. Mouth/chin left	☐
5. Neck right	☐	11. Neck left	☐
6. Temporomandibular joint right	☐	12. Temporomandibular joint left	☐

Gothenburg Trismus Questionnaire (GTQ 2 191028). Used by permission © Finizia, Rydén, Johansson
Sahlgrenska Academy, Gothenburg University, Sweden

Below you find some questions about training and the device you might be using. Please read each question carefully and answer by marking the alternative that best applies to you.

24. Are you training to improve your ability to open your mouth (with a mouth stretching device, using your fingers to stretch or something else)?

☐ Yes (1) ☐ No (0) If yes what kind of training do you perform?

(Training with a mouth stretching device, using your fingers to stretch or something else)

____

	**Not at all**	**A little**	**Moderately**	**Quite a bit**	**Very much**
	**1**	**2**	**3**	**4**	**5**
25. Is the training/training device used to improve your mouth opening ability *uncomfortable to use*?	☐	☐	☐	☐	☐
26. Is it *physically exhausting* to train/use the training device to improve your mouth opening ability?	☐	☐	☐	☐	☐
27. Does it *take a lot of time* doing the mouth opening exercises?	☐	☐	☐	☐	☐
28. Do you find the exercises *efficient, that is - do they help?*	☐	☐	☐	☐	☐

29. On average, how many times a day do you practise?

**Not at all**	**Once a day**	**Twice a day**	**Three times a day**	**Four times a day**	**Five or more times a day**
☐0	☐1	☐2	☐3	☐4	☐5

Gothenburg Trismus Questionnaire (GTQ 2 191028). Used by permission © Finizia, Rydén, Johansson
Sahlgrenska Academy, Gothenburg University, Sweden
